# Reconstruction of 3D genome architecture via a two-stage algorithm

**DOI:** 10.1186/s12859-015-0799-2

**Published:** 2015-11-09

**Authors:** Mark R. Segal, Henrik L. Bengtsson

**Affiliations:** Division of Bioinformatics, Department of Epidemiology and Biostatistics, University of California, 550 16th Street, San Francisco, 94158 CA USA

**Keywords:** Chromatin conformation capture, Multi-dimensional scaling, Procrustes transformation, Downsampling

## Abstract

**Background:**

The three-dimensional (3D) configuration of chromosomes within the eukaryote nucleus is an important factor for several cellular functions, including gene expression regulation, and has also been linked with cancer-causing translocation events. While visualization of such architecture remains limited to low resolutions, the ability to *infer* structures at increasing resolutions has been enabled by recently-devised chromosome conformation capture techniques. In particular, when coupled with next generation sequencing, such methods yield an inventory of genome-wide chromatin contacts or interactions. Various algorithms have been advanced to operate on such contact data to produce reconstructed 3D configurations. Studies have shown that these reconstructions can provide added value over raw interaction data with respect to downstream biological insights. However, only limited, low-resolution reconstructions have been realized for mammals due to computational bottlenecks.

**Results:**

Here we propose a two-stage algorithm to partially overcome these computational barriers. The central idea is to initially utilize existing reconstruction techniques on an individual chromosome basis, using *intra*-chromosomal contacts, and then to relatively position these chromosome-level reconstructions using *inter*-chromosomal contacts. This two-stage strategy represents a natural approach in view of the within- versus between- chromosome distribution of contacts. It can increase resolution ≈ 20 fold for mouse and human. After describing the algorithm we present 3D architectures for mouse embryonic stem cells and human lymphoblastoid cells. We evaluate the impact of several factors on reconstruction reproducibility and explore a variety of sampling schemes. We further analyze replicate data at differing resolutions obtained from recently devised *in situ* Hi-C assays. In all instances we demonstrate insensitivity of the whole-genome 3D reconstruction obtained by the two-stage algorithm to the sampling strategy used.

**Conclusions:**

Our two-stage algorithm has the potential to significantly increase the resolution of 3D genome reconstructions. The improvements are such that we can progress from 1 Mb resolution to 100 kb resolution, notable since this latter value has been identified as critical to inferring topological domains in analyses performed on the contact (rather than 3D) level.

## Background

The three-dimensional (3D) configuration of chromosomes within the eukaryote nucleus is important for several cellular functions, including gene expression regulation and epigenetic patterning [1], and has also been linked to translocation events and cancer driving gene fusions [2, 3]. While visualization of such architecture remains limited to low resolutions (due to compaction, dynamics and scale), the ability to *infer* structures at high resolution has been enabled by recently-devised assays derived from chromosome conformation capture (3C) techniques [4]. In particular, when coupled with next generation sequencing, such methods (hereafter termed *Hi-C* [5, 6]) yield an inventory of genome-wide chromatin interactions which, in turn, form the basis for reconstructing 3D configurations [7, 8].

There have been ongoing improvements in assay design [9, 10]. These include use of greater sequencing depths that enable higher resolution analyses [11], notable for supporting the elicitation of *mammalian* topological domains – highly self-interacting regions – when between-loci interactions (contact counts) are binned at sizes less than 100 kilobases (kb), in contrast to earlier analyses conducted at the megabase (Mb) level [5]. More recently, use of *in situ* Hi-C [12] has facilitated analyses at 1 kb resolution, refining topological domains into smaller contact domains of median length 185 kb that were previously undetectable. Similarly, companion preprocessing and normalization tools for Hi-C data have emphasized handling higher resolutions [13]. However, the suite of 3D reconstruction algorithms has not kept pace with these resolution improvements. In fact, computational bottlenecks have largely precluded high-resolution, whole-genome mammalian reconstructions. This is consequential beyond the abovementioned *intra*-chromosomal identification of topological domains. It has been shown for simpler organisms that such 3D reconstructions provide added-value over raw interaction data with respect to downstream identification of co-localization (both intra- and *inter*- chromosomal) of genomic landmarks and functional groups, with the human malaria parasite *P. falciparum* [14] and the yeast *S. cerevisiae* [15] being examples. Moreover, superposing functional outcomes, such as gene expression [14] or ChIP-Seq peaks (Capurso D, Bengtsson H, Segal MR: Identifying hotspots in functional genomic data superposed on 3D chromatin configuration reconstructions. Submitted.), on 3D reconstructions have facilitated biological insights unobtainable from contact maps.

Here we advance a two-stage algorithm that seeks to reduce these computational barriers. For mouse and human, resolution improvements on the order of 20 fold are attainable. We note that many of the previously proposed methods can serve as primitives for our approach. Much of the effort surrounding these previously developed reconstruction methods is comparative – so as to differentiate between approaches and establish superiority of the technique under consideration. This has given rise to simulations of selective scope and/or use of – often out of necessity – sub-optimal real-data comparisons. Accordingly, we view this emphasis as misplaced and expand on these issues in the ‘Discussion’. Our objective here is to simply offer proof-of-principle for a strategy whereby existing algorithms can be deployed to greater effect and, in particular, to enable reconstruction of 3D genome structures at improved resolutions.

## Methods

The result of a Hi-C experiment, following important preprocessing and normalization steps [13, 16–19], is the *contact map*, a symmetric *n*×*n* matrix *F* = [*F*_*ij*_] of contact counts between *n* (generally binned) genomic loci *i, j* on a genome-wide basis. This matrix can be exceedingly sparse with many zero entries even after binning. The 3D genome reconstruction problem is then to obtain a 3D point configuration, with a one-to-one correspondence between points and genomic loci, such that the resulting pairwise inter-point distances best recapitulate the corresponding frequencies. We operationalize “best recapitulate” below. Many approaches have been proposed to tackle this problem, with broad distinction [8, 20] between optimization/consensus and probabilistic/ensemble methods, although these can overlap. Our focus here is on the former. A common first step in these reconstruction algorithms (e.g. [6, 8, 9, 14, 24–27]) is conversion of the frequency matrix into a distance matrix *D*=[*D*_*ij*_]. This is often followed by a second step which solves the graph realization [28] or multi-dimensional scaling (MDS, [29]) problem: position points (corresponding to genomic loci) in 3D so that the resultant interpoint distances best conform to the distance matrix. We briefly describe aspects of each of these components in order to position our two-stage algorithm.

### Converting contact frequencies to distances

A range of methods have been used for transforming frequencies into distances. At one extreme, in terms of invoking and imposing biological assumptions, are methods that proceed by relating observed intra-chromosomal contacts to genomic distances and then ascribing *physical* distances based on organism specific findings on chromatin packing [6] or ostensibly well-defined [21, 22] relationships between genomic and physical distances for crumpled polymers [14]. Such distances inform the subsequent optimization step as they allow for incorporation of known biological constraints that can be expressed in terms of physical separation. However, obtaining physical distances requires strong assumptions with the conversion being dependent on organism [23], resolution [7] and cell cycle [30]; see also [8].

At the other extreme are methods devoid of biology-based inputs. Lesne et al. [24] assign a weighted link of 1/*F*_*ij*_ between loci *i* and *j* and then arrive at a distance matrix by computing the shortest (weighted) path between all loci pairs. The shortest path is obtained using the $\mathcal {O}(n^{3})$ Floyd-Warshall algorithm. The associated algorithm “shortest-path reconstruction in 3D (ShRec3D)" then uses MDS to obtain a reconstruction. ShRec3D is purportedly insensitive to link weighting and enjoys good overall performance speed as further described in the ‘Discussion’.

An intermediary strategy is represented by the methodology of Zhang et al. [7]. This approach, *ChromSDE*, (i) invokes a power law relationship between spatial distances and contact frequencies: *D*_*ij*_=(*F*_*ij*_)^−*α*^ if *F*_*ij*_>0; *D*_*ij*_=*∞* if *F*_*ij*_=0, (ii) uses weighted, penalized MDS to obtain points in 3D conforming to *D*, and (iii) alternates between (i) and (ii) using golden section search to optimize for *α*. The power law prescription derives from empiric and theoretic work on biopolymers in general and DNA in particular [5, 23]. Our subsequent analyses use ChromSDE as a building block toward obtaining whole genome architecture for mouse and human. However this choice, while motivated by the good properties of ChromSDE, is not critical – alternate techniques can be readily used.

### Graph realization / multi-dimensional scaling formulation

We seek a 3D configuration $X = \{\vec {x}_{1},\ldots,\vec {x}_{n}\}; \vec {x}_{i} \in R^{3}$ that best fits the distance matrix *D* according to weighted, penalized graph realization or MDS criteria as framed by Zhang et al. [7]:

(1) $$ {\small{\begin{aligned} {}\! \! \! \! \! \! \min_{\{\vec{x}_{1},\ldots,\vec{x}_{n} | \sum \vec{x}_{i} =0 \}} \! \sum_{\{i,j | D_{ij} < \infty\}} \! \!\omega_{ij} \cdot\! (\| \vec{x}_{i} \,-\, \vec{x}_{j} \| \!\,-\, D_{ij})^{2} \,-\, \lambda \! \! \sum_{\{i,j | D_{ij} = \infty\}} \! \! \| \vec{x}_{i} \,-\, \vec{x}_{j} \|^{2} \end{aligned}}}  $$

(2) $$ {\small{\begin{aligned} {}\! \! \! \! \! \! \min_{\{\vec{x}_{1},\ldots,\vec{x}_{n} | \sum \vec{x}_{i} =0 \}} \! \sum_{\{i,j | D_{ij} < \infty\}} \! \!\omega_{ij} \cdot\! \left| \!\| \vec{x}_{i} \,-\, \vec{x}_{j} \|^{2} \,-\, D_{ij}^{2} \right| \,-\, \lambda \! \! \sum_{\{i,j | D_{ij} = \infty\}} \! \! \| \vec{x}_{i} \,-\, \vec{x}_{j} \|^{2} \end{aligned}}}  $$

(3) $$ {\small{\begin{aligned} {}\! \! \! \! \! \! \min_{\{\vec{x}_{1},\ldots,\vec{x}_{n} | \sum \vec{x}_{i} =0 \}} \! \sum_{\{i,j | D_{ij} < \infty\}} \! \!\omega_{ij} \!\cdot\! (\| \vec{x}_{i} \,-\, \vec{x}_{j} \|^{2} \,-\, D_{ij}^{2}) \,-\, \lambda \! \! \sum_{\{i,j | D_{ij} = \infty\}} \! \! \| \vec{x}_{i} \,-\, \vec{x}_{j} \|^{2}  \end{aligned}}}  $$

The purpose of the weights, *ω*_*ij*_, in each criterion, is to mitigate the influence of large *D*_*ij*_ values which correspond to small *F*_*ij*_ – it is for such small contact counts that experimental data is least reliable. Common choices for *ω*_*ij*_ include $D_{\textit {ij}}^{-1}$ [7] and $D_{\textit {ij}}^{-2}$ [8]. Similarly, the common penalty (second) term maximizes pairwise distances for the many loci without any contacts for which the weighting approach is undefined without introducing fudge factors or filtering schemes [24]. Each criterion corresponds to a nonconvex nonlinear optimization problem that is NP hard. By relaxing the solution space for each $\vec {x}_{i}$ from *R* ^3^ to *R* ^*n*^, Zhang et al. [7] demonstrate how criteria (2) and (3) can be recast as convex semidefinite programming (SDP) problems via the kernel *K* for *X* ($K_{\textit {ij}} = \vec {x}_{i} \cdot \vec {x}_{j}$) for which it is possible to obtain global optima in polynomial time. Given a solution kernel, coordinates in 3D can be recovered by projection based on the first three eigenvalues and eigenvectors of its spectral decomposition. Importantly, they detail the unique localizability condition [28] which ensures that, in noise-free settings, the SDP solution is exact. This property is not shared by other approaches to 3D reconstruction.

Their resulting algorithm, *ChromSDE*, provides a compelling approach to 3D structure elicitation problems, possessing several desirable attributes including: (i) fast (polynomial time) solutions; (ii) guaranteed recovery of the correct structure in the noise-free case; (iii) adaptive estimation of the power law index *α*; and (iv) provision of a consensus index that indicates whether the Hi-C data derives from a single structure versus a mixture of structures.

The main limitation of ChromSDE, which impacts other reconstruction approaches, is simply computational: since distance matrices scale as the square of the number of loci, problem size becomes an issue before informative resolutions (<100 kb for mouse, human [11]) can be tackled. While ChromSDE employs a sophisticated, recently developed algorithm [31] for convex quadratic SDPs (as result from relaxation of (3)) that supports much larger problems (*n*≈3000 loci) than general SDP solvers (*n*≈200 loci), this resolution is still an order of magnitude less than the desired target resolutions for human and mouse. Such resolution bottlenecks are computational and not assay related, with contemporary sequencing capacity producing reliable Hi-C data at high resolution. Accordingly, as has been noted [8], ChromSDE only provides solutions for individual chromosomes. As we describe next, these can serve as the first step toward a whole-genome reconstruction.

### Whole genome solution via a two-stage algorithm

Our two-stage approach is based on the above computational considerations coupled with properties of interaction frequencies *F*_*ij*_. By virtue of chromatin contiguity, and the tendency for interphase chromosomes to occupy distinct territories within at least the human nucleus [32], there is a preponderance (≈ 15−20 fold) of intra-chromosomal contacts compared to inter-chromosomal contacts. So, at stage one, we exploit the bulk of the frequency signal by obtaining individual chromosomal configurations using ChromSDE or an alternate reconstruction procedure. We are now faced with the problem of determining the *relative positioning* of these individual chromosomes in order to obtain a whole-genome reconstruction. For this we turn to the inter-chromosomal contacts, but due to the above mentioned computational considerations, we can only combine these with a *selection* of intra-chromosomal points. While intra-chromosomal contacts provide the bulk of the counts, inter-chromosomal contacts provide the bulk of the interacting *pairs*: ≈10 fold more for mouse and human. By using these to *stitch* together the individual chromosome solutions, we arrive at a whole-genome reconstruction that effectively uses all the data. Details for this program are as follows.

Let $X^{k} = \{\vec {x}_{i}\}; i=1,\ldots,n_{k}$ be the ChromSDE solution (i.e. 3D coordinates) for chromosome *k* (*k*=1,…,*K*). We sample *m*_*k*_ points without replacement from *X* ^*k*^ and discuss strategies for effecting the sampling and prescribing *m*_*k*_ or $m = \sum _{k} m_{k}$ in both the ‘Results’ and ‘Discussion’. Designate the sampled points as $\tilde {X}^{k}$. Let $\tilde {D}^{k}$ be the distance matrix obtained by computing all pairwise Euclidean distances among the $\tilde {X}^{k}$ sampled points; these $\tilde {D}^{k}$ capture intra-chromosomal distances based on the ChromSDE reconstruction. To obtain inter-chromosomal distances with respect to the sampled $\vec {x}_{i} \in \tilde {X}^{k}, \vec {x}_{j} \in \tilde {X}^{k'}$ we compute $\tilde {D}_{\textit {ij}} = (\tilde {F}_{\textit {ij}})^{- \sqrt {\alpha _{k} \cdot \alpha _{k'}}}$ where $\alpha _{k}, \alpha _{k'\phantom {\dot {i}\!}}$ are the power law indices obtained for chromosomes *k, k* ^′^ from the ChromSDE solutions and $\tilde {F}_{\textit {ij}}$ is the interaction frequency between the loci corresponding to $\vec {x}_{i}$ and $\vec {x}_{j}$. If $\tilde {F}_{\textit {ij}} = 0$, we can either replace with a small pseudo count [25] or employ penalization as per (1)–(3).

The $\tilde {D}^{k}$ serve as block diagonal matrices of an overall *m*×*m* distance matrix $\tilde {D}^{*}$ for which the above $\tilde {D}_{\textit {ij}}$ provide off block diagonal elements. Now we can re-apply MDS/graph realization algorithms to $\tilde {D}^{*}$ to find points in 3D that best recapitulate the *hybrid* distance matrix that combines actual Euclidean distances from the within chromosome ChromSDE solutions with power-law inferred between chromosome distances. Let the resulting solution (3D coordinates) be $\tilde {X}^{*}$. This represents a whole-genome (all *K* chromosomes) reconstruction, but only for the *m* sampled loci. To obtain a configuration for all loci we use Procrustes transformation [29, 33], on a per chromosome basis, to rigidly map the original $\tilde {X}^{k}$ to the corresponding points in $\tilde {X}^{*}$. The Procrustes analysis, here implemented using the R package vegan [34], provides the rotation matrix and translation vector for effecting the mapping; these are then applied to each original ChromSDE solution, *X* ^*k*^, to obtain a full-resolution, whole-genome solution. Procrustes analysis also provides an optional scaling factor for resizing mapped solutions. We have utilized such rescaling throughout. While such rescaling may be less critical for methods that, unlike ChromSDE, estimate a scale factor as part of the conversion of counts to distances, it ought nonetheless be applied in view of the lack of scale invariance of measures (like RMSD) used to assess agreement between reconstructions as described next in ‘Results’.

## Results

### Mouse and human data at 1 Mb resolution

**Fig. 1:**
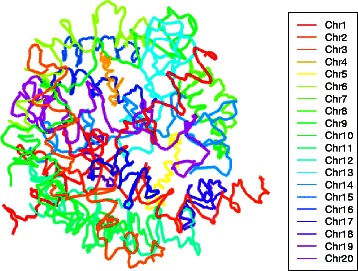
3D reconstruction for mouse embryonic stem cells. Whole genome architecture, at 1 Mb resolution, from applying the two-stage reconstruction algorithm with 10 % equi-spaced sampling to mESC cell line data [11]

**Fig. 2:**
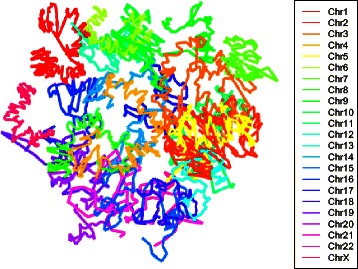
3D reconstruction for human lymphoblastoid cells. Whole genome architecture, at 1 Mb resolution, from applying the two-stage reconstruction algorithm with 10 % equi-spaced sampling to human GM06990 cell line data [5]

We initially applied the two-stage strategy described above to public Hi-C data from two differing cell lines: mouse embryonic stem cells (mESC) [11] and human lymphoblastoid cells (GM06990) [5]. For each of these cell lines two datasets were available at 1 Mb resolution corresponding to two restriction enzyme (RE) digests (HindIII and NcoI). In all instances, interaction frequencies were normalized using *hicpipe* [16]. Reconstructions for mESC and GM06990 cell lines for HindIII digestion using the two stage algorithm are depicted in Figs. 1 and 2, respectively. In these reconstructions, the stage two sampling scheme consisted of taking 10 equi-spaced points for each chromosome. The weights used for MDS in both stage one and stage two were *ω*_*ij*_=1/*D*_*ij*_.

Evaluating *accuracy* of 3D genome reconstructions is challenging. Fluorescence *in situ* hybridization (FISH) techniques have served as a “gold standard” for 3D reconstructions [5, 9, 10, 14]. However, the number of FISH landmarks available is small and so this approach is limited with respect to arbitrating between reconstructions at the target resolutions. More importantly, a crucial feature of FISH data generated to date is that the landmarks all derive from *individual* chromosomes. This pertains even for 3D genome reconstruction methods that integrate FISH-based calibration into their algorithms [25]. Since our two-stage approach starts with obtaining 3D reconstructions of individual chromosomes using any *existing* method, and then determines their relative positioning, this data can only be used for evaluating the (existing) algorithms on an individual chromosome basis.

So, we turn to widely-used assessments of *reproducibility* [7, 8, 14, 36, 37] to examine the impact of sampling schemes, and other facets of the two-stage algorithm, by measuring agreement (“closeness”) between reconstructions obtained under a variety of conditions. Let *X*_*s*_,*X*_*t*_ be *n*×3 matrices with rows being the 3D coordinates for the *n* (common) loci resulting from two such reconstructions. Two (implicitly correlated) measures of agreement between *X*_*s*_ and *X*_*t*_ are commonly used: root mean squared deviation (RMSD) and distance error. Configurations that only differ by a reflection, rotation, translation and scaling (*reflection similarity shape*, [35]) are deemed equivalent. The RMSD closeness of *X*_*s*_ and *X*_*t*_ is then measured by how far apart corresponding points are, after optimizing for these allowed transformations. Such optimization is effected via Procrustes analysis. As mentioned, this includes always estimating a rescaling parameter as part of the Procrustes transformation which mitigates the lack of scale invariance of the RMSD criterion. Distance error avoids the need for transformation by comparing corresponding within-reconstruction distances. Let $d(\vec {x}_{i}^{s},\vec {x}_{j}^{s})$ be the Euclidean distance between positions $\vec {x}_{i}^{s}, \vec {x}_{j}^{s}$ of rows (loci) *i, j* of *X*_*s*_ and similarly for *X*_*t*_. Then distance error is given by $\sqrt {\sum _{i <j}^{n} (d(\vec {x}_{i}^{s},\vec {x}_{j}^{s}) - d(\vec {x}_{i}^{t},\vec {x}_{j}^{t}))^{2}}$. However, as formulated, distance error is also not scale invariant. Following [37] we use the scale invariant version obtained by substituting $d^{*}(\vec {x}_{i}^{s},\vec {x}_{j}^{s}) = d(\vec {x}_{i}^{s},\vec {x}_{j}^{s})/\sum _{i<j}d(\vec {x}_{i}^{s},\vec {x}_{j}^{s})$ for $d(\vec {x}_{i}^{s},\vec {x}_{j}^{s})$ and similarly for $d(\vec {x}_{i}^{t},\vec {x}_{j}^{t})$.

The experimental design for examining reconstructions under differing conditions for the mESC data consisted of a complete cross between *sampling scheme* (3 differing equi-spaced selections) ×*restriction enzyme* (HindIII, NcoI) ×*stage two distance specification* (hybrid, non-hybrid) for a total of 12 conditions. Additional reconstructions utilized non-metric MDS at stage two via the smacof R package [38], however, convergence/computational difficulties resulted in very few solutions. The hybrid category for stage two distance specification refers to the blending of within-chromosome Euclidean distances with between-chromosome power law derived distances as detailed in the preceding section. The non-hybrid category pertains to using the same sampling scheme as for the hybrid approach but using power law derived distances both within and between chromosomes. Thus, this factor addresses putative gains afforded by the two stage algorithm. The sampling schemes utilized every 10th bin from differing start positions. More sophisticated alternatives are described below and in the ‘Discussion’.

The 12 conditions give rise to ${12 \choose 2} = 66$ pairwise comparisons for which we obtain (standardized) distance error and RMSD measures of agreement. We partition the condition defining factors according to between- and within-level comparisons and fit separate regression models for the two (outcome) measures of agreement. Results are presented in Tables 1 (distance error) and 2 (RMSD). For both measures we observe highly significant differences for between RE comparisons versus within RE comparisons, with the within RE structures being closer than their between RE counterparts. There is no statistical difference between the two within RE categories. Similarly, for distance error, between hybrid and power-law distance comparisons are significantly different than the corresponding within distance type comparisons, again there being no statistically significant distinction between the two within distance type categories. It is notable that for both measures there are no differences between the sampling schemes. While the extent of sampling schemes examined is modest, this null result provides some reassurance that the proposed two-stage approach, with its sampling component, can produce concordant whole genome 3D reconstructions.

**Table 1:** Regression estimates for standardised distance error

	Estimate	StdError	*t*-value	Pr(>|*t*|)
Within HindIII	referent			
Between HindIII & NcoI	1.340e-05	2.375e-06	5.639	5.83e-07
Within NcoI	–3.277e-06	2.788e-06	–1.175	0.245
Within hybrid distances	referent			
Between hybrid & power-law	1.302e-05	2.375e-06	5.482	1.04e-06
Within power-law distances	–2.222e-06	2.788e-06	–0.797	0.429
Within sample 1	referent			
Between samples 1 & 2	4.609e-07	3.686e-06	0.125	0.901
Between samples 1 & 3	3.561e-07	3.686e-06	0.097	0.923
Within sample 2	5.851e-06	4.408e-06	1.328	0.190
Between samples 2 & 3	–4.143e-07	3.686e-06	–0.112	0.911
Within sample 3	–3.388e-06	4.408e-06	–0.769	0.445

**Table 2:** Regression estimates for RMSD

	Estimate	StdError	*t*-value	Pr(>|*t*|)
Within HindIII	referent			
Between HindIII & NcoI	0.0179	0.0085	2.101	0.040
Within NcoI	–0.0129	0.0100	–1.297	0.200
Within hybrid distances	referent			
Between hybrid & power-law	0.0113	0.0085	1.320	0.192
Within power-law distances	0.0024	0.0100	0.246	0.806
Within sample 1	referent			
Between samples 1 & 2	–0.0026	0.0132	–0.198	0.844
Between samples 1 & 3	0.0004	0.0132	0.033	0.974
Within sample 2	0.0111	0.0158	0.699	0.487
Between samples 2 & 3	–0.0037	0.0132	–0.276	0.783
Within sample 3	0.0076	0.0158	0.481	0.632

We next sought to further assess this finding by performing a more extensive exploration of sampling schemes as applied to the human GM06990 lymphoblastoid cell line data. Here we used three sampling schemes: equi-spaced, binned, and first principal components each at 10 sampling fractions (0.1,0.2,…,1.0). Equi-spaced sampling selects evenly spaced bins according to the prescribed sampling proportion. Instead of selection of individual bins, aggregation combines bins so as to attain the specified sampling proportion. The principal component scheme is an attempt to go beyond (linear) genomic coordinate based sampling and to utilize (minimal) 3D information. For each individual chromosome 3D reconstruction the first principal component of the attendant points is obtained. The points are projected onto this component and sampling proceeds, according to prescribed proportion, along this component. This represents a crude, albeit computationally straightforward, way to capture local 3D density as part of the sampling scheme. Comparisons were effected by specifying a referent reconstruction from which the RMSD of all other reconstructions was computed. Use of such a common referent allows for comparisons across settings in view of the previously mentioned concern of RMSDs not being scale invariant.

**Fig. 3:**
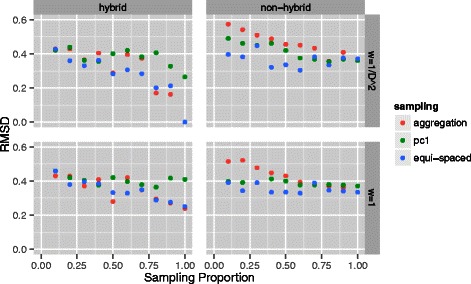
RMSDs for a series of sampling proportions and schemes. RMSD comparisons across a range (0.10,0.20,…,1.0) of second stage sampling proportions and schemes (equi-spaced, aggregated and first principal component based) for reconstructions from human GM06990 lymphoblastoid cell lines [5]. Hybrid and non-hybrid algorithms and MDS weighting options are also contrasted. Comparisons are performed with respect to a referent reconstruction based on equi-spaced sampling at a proportion of 1.0, under the non-hybrid algorithm with inverse squared distance weighting

Results are depicted in Fig. 3. The referent reconstruction was chosen to be that based on equi-spaced sampling at a proportion of 1.0, under the hybrid algorithm with inverse squared distance weighting. When this reconstruction is compared against itself, an RMSD of 0 is obtained as seen in the upper left panel. Some findings are readily apparent. First, results are reasonably stable with respect to sampling proportions, with evidence of improved agreement (smaller RMSDs) corresponding to larger fractions. Second, there are no consistent or pronounced differences between sampling schemes. Third, the hybrid algorithm tends to produce smaller RMSDs than the non-hybrid algorithm across all sampling proportions. Fourth, concordant RMSD values are obtained regardless of whether weighted ($\omega _{\textit {ij}} = 1 / D_{\textit {ij}}^{2}$) or unweighted (*ω*_*ij*_=1) MDS is used for stage two relative positioning. Identical findings pertained for other choices of the referent reconstruction. So, in summary, this collection of results further supports the conclusion that whole genome 3D reconstructions are not overly influenced by the choice of sampling scheme and/or fraction or, indeed, other aspects of the second stage repositioning step. These findings pertained down to a sampling fraction of 5 %. Such a result is critical for the proposed two-stage strategy to have any potential. As we detail further in the ‘Discussion’, this assessment is supported by recent work that approached sampling and 3D reconstruction from a different perspective [26].

### Improved resolution and replication human data

A more comprehensive evaluation of resolution and replication aspects was pursued using a richer dataset. Rao et al. [12] deploy their *in situ* Hi-C assay to comprehensively map genome-wide chromatin contacts in a variety of cell lines, at several resolutions, and, importantly, to examine replicates thereof. Here we utilize their data from GM12878 B-lymphoblastoid cells at 1 Mb and 500 kb resolutions, each from primary and replicate studies. These datasets represent pools over individual experiments: 18 for the primary series and 11 for the replicate series. We use unnormalized, filtered versions that restrict to read pairs with mapping Q scores greater than or equal to 30. Stage one individual chromosome reconstructions used MDS with *ω*_*ij*_=1/*D*_*ij*_. We use the same ten equi-spaced sampling fractions as above and again make recourse to a referent reconstruction selected at the maximal sampling proportion (i.e. complete data) for the primary series. Additionally, we computed RMSDs between primary and replicate series at each sampling fraction without use of a global referent (not shown).

**Fig. 4:**
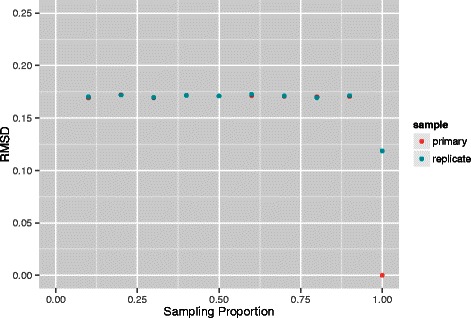
RMSD comparisons between replicates at 1 Mb resolution. Comparisons across a range (0.10,0.20,…,1.0) of second stage equi-spaced sampling proportions for reconstructions from primary and replicate human GM12878 B-lymphoblastoid cell line pools [12] at a resolution of 1 Mb. The referent reconstruction is based on a proportion of 1.0 for the primary series. Overplotting obscures coincident points at some sampling proportions

**Fig. 5:**
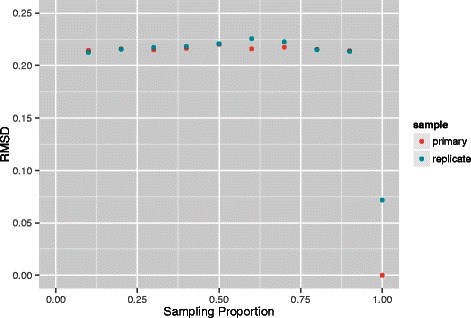
RMSD comparisons between replicates at 500 kb resolution. Comparisons across a range (0.10,0.20,…,1.0) of second stage equi-spaced sampling proportions for reconstructions from primary and replicate human GM12878 B-lymphoblastoid cell line pools [12] at a resolution of 500 kb. The referent reconstruction is based on a proportion of 1.0 for the primary series. Overplotting obscures coincident points at some sampling proportions

Results are displayed in Figs. 4 and 5. Once again, we see invariance with respect to sampling proportion. Moreover, at both resolutions, distance as measured via RMSD to the referent is almost identical for the primary and replicate studies at all sampling fractions and these values are not substantially increased over the RMSD for the replicate series when no downsampling is employed. While examination of per sampling fraction primary versus replicate comparisons, without use of a global referent at both 1 Mb and 500 kb resolutions revealed no systematic trends, there was no indication of deteriorated performance at low sampling fractions. We also made comparisons between these resolutions. These were performed by *thinning* a given 500 kb reconstruction, for a particular sampling fraction, so that the genomic loci corresponding to each 3D point of the reconstruction were also represented in the 1 Mb reconstruction at that same sampling fraction. The thinning essentially amounts to considering every other point. Procrustes transformation was then used to align these reconstructions and the attendant RMSD obtained. These RMSDs were very small across the suite of sampling fractions ranging from 1.6 ×10^−3^ to 4.0 ×10^−3^. This good agreement in part reflects the fact that the data underlying the 1 Mb reconstructions is arrived at by binning the 500 kb data, as opposed to being independently generated.

We evaluated how well the coordinates corresponding to *each* bin in the stage one individual chromosome reconstructions matched their counterparts in the merged whole-genome reconstruction as obtained by the second stage. Despite the whole-genome reconstruction being obtained by rigid Procrustes transformation, these point sets do not necessarily coincide since this transformation is determined solely by the *sampled* bins. In brief, agreement as measured by RMSD was comparable across all sampling proportions, this holding for both primary and replicate data series at both 500 kb and 1 Mb. For a given sampling fraction and resolution, there was excellent agreement between primary and replicate data RMSDs on a per chromosome basis. There was a tendency toward greater between-chromosome RMSD variability at small (≤0.3) sampling proportions as compared with large (≥0.7) proportions.

Note that sampling fractions employed extend to 1.0 even for data at 500 kb resolution. That is, we were able to perform stage two MDS on a distance matrix *D* with row and column dimension of approximately *m*=3×10^9^/500×10^3^=6,000. An *m*×*m* distance matrix *D* occupies *m*×*m*×size of (double)=*m*×*m*×8 bytes of RAM, which for the 500 kb resolution comes to 275 MB of RAM (1 MB = 1024 ^2^ bytes). Roughly allowing for memory complexity of the smacof algorithm, coupled with the need to utilize an *m*×*m* weight matrix, by introducing a factor of 10 results in memory requirements of 2.7 GB of RAM, which can readily be handled. However, in attempting to pursue 3D reconstructions at 100 kb resolution we encounter runtime (as opposed to memory) challenges. Specifically, while we can obtain reconstructions at sampling fractions of 5, 10, 15, 20, 30 and 40 % in respectively 5.0, 8.0, 21, 56, 117, and 480 h on a 2.6 GHz Opteron processor with 512 GB of memory. We comment further on memory and computational complexity in the context of higher resolution *in situ* Hi-C data in the 1.

## Discussion

The purpose of this article was to demonstrate proof-of-principle for a two-stage approach to obtaining whole genome 3D reconstructions from Hi-C assays. The advantages of such a strategy include (i) effective use of both within chromosome (majority of interaction counts) and between chromosome (majority of interaction pairs) contacts; (ii) the ability to use existent reconstruction tools, especially for the first stage generation of individual chromosome solutions; and (iii) the potential to obtain higher resolution whole genome reconstructions. As noted, this latter point is consequential with respect to eliciting topological and contact domains in mammalian genomes [11, 12]. The two-stage reconstruction method could potentially be improved by employing more sophisticated sampling schemes. These could make recourse to targeting loci with large inter-chromosomal counts or be based on curvature summary measures for the individual chromosome solutions. Moreover, a variety of sampling schemes could be deployed and a consensus (averaged) structure reported after performing Procrustes alignment. The regression-based testing of between versus within sample variation, as utilized for the comparisons presented in the ‘Results’, could be used as a precursor determinant as to whether such averaging was justified, analogous to the consensus index [7].

For demonstration purposes we have, in part, used ChromSDE for stage one reconstructions of individual chromosomes because of its desirable characteristics. Two recent papers purportedly show relatively poor performance of ChromSDE; accordingly we make some brief comments on these assessments. Varoquaux et al. [8] use MDS to provide an initial 3D reconstruction then refine this configuration using Poisson regression. In making comparisons between differing reconstruction approaches, they comment that “ChromSDE does not infer the relative position of chromosomes.” Our two-stage algorithm provides such relative positioning thereby expanding the scope and utility of ChromSDE. Moreover, based on simulation studies, [8] contend that ChromSDE performs relatively poorly in low signal-to-noise settings. However, the simulations were based on data generated according to their Poisson model and employed a fixed *α* value conforming to this model. Such a framework deprives ChromSDE from demonstrating putative advantages associated with estimating *α*. A more complete comparison would result from applying the Poisson model to data generated with a misspecified *α* value.

In evaluating ShRec3D, Lesne et al. [24] also make comparisons with ChromSDE, again based on simulation. While the methods are comparable with respect to accuracy, ChromSDE is deemed worse with respect to both problem size capability and computational time. Again, these are aspects wherein our proposed two-stage algorithm can extend applicability of ChromSDE. Further, contrary to [7], Lesne et al. contend that the choice of *α* is not critical. As they note, weak or vanishing contact frequencies do not contribute to inferred distances, since shortest path constructions will avoid corresponding links (of large or infinite lengths). Accordingly, ShRec3D involves an implicit filtering. Since 3D solutions are also obtained via MDS it is presumably the degree of this filtering that is responsible for its improved problem size and compute time characteristics.

Recent work by Diament and Tuller [26] also employs sampling as a precursor to 3D genome reconstruction. For *S. cerevisiae* they demonstrate that even under sparse samplings, down to the 5 % level, the resultant reconstructions are concordant with the original reconstruction in terms of co-localization of various genomic landmarks and functional groups. This concordance is used to suggest the possibility of reducing the scale of Hi-C experimentation, if 3D reconstruction is used to analyze the data. As such, their findings are consistent with our results that show that sampling, also down to the 5 % level, does not impact the 3D reconstruction itself. Accordingly, our two-stage approach is able to realize 20-fold improvements in resolution.

The mouse and human data analyzed herein are diploid. To date, 3D whole-genome reconstructions based on Hi-C data from diploid organisms implicitly treat homologous chromosome pairs as coincident (i.e. as occupying roughly the same position, relative to inter-chromosomal positioning) [7, 8, 24]. The reasonableness of such an assumption is unclear. The emergence of high resolution *in situ* Hi-C assays [12] has enabled phasing of Hi-C maps. This derives from having sufficient reads overlap SNPs so that contacts can be assigned to specific chromosomal homologs. At present, the resolutions needed for such phasing are such that computational bottlenecks, even with our two-stage algorithm, preclude 3D genome reconstruction on a diploid level. Even though the disambiguation provided by phasing shows that, for autosomes, maternal and paternal homologs exhibit very similar inter- and intra- chromosomal contact profiles (Pearson’s *R*>0.998) and, accordingly, very similar between loci distances, this does not in itself imply that are homologous pairs are roughly coincident in view of results from computational geometry [39]. We note that for a *near*-haploid chronic leukemia cell line (KBM7) Ay et al., [27] provide a diploid reconstruction.

Contact data at 1 kb resolution from *in situ* Hi-C assays will challenge existing 3D reconstruction algorithms on both memory and computational complexity grounds. For example, 10 % downsampling of whole genome human 1 kb resolution data will still yield a distance matrix requiring ∼670 GB of RAM. Accordingly, even obtaining individual chromosome solutions will require downsampling and/or algorithmic refinements (*cf.* [40, 41]). However, once these are generated, the two-stage approach developed here will again be valuable in eliciting whole genome 3D architecture.

## Conclusions

The proposed two-stage algorithm offers a promising technique for obtaining whole genome 3D reconstructions from Hi-C assays. By sampling 3D coordinates from individual chromosome solutions (obtained using any existing method), and deploying inter-chromosomal contacts to relatively position these solutions, it efficiently leverages contact data enabling substantial improvements in resolution.
